# Supplementary material for: Hybrid Models and Biological Model Reduction with PyDSTool
Source: PLoS Comput Biol. 2012 Aug 9;8(8):e1002628. doi: 10.1371/journal.pcbi.1002628 (PMC3415397; doi:10.1371/journal.pcbi.1002628)
Supplement: Text S4 — Complete source code for the PyDSTool package (version 0.88.120504). Includes API documentation and help files linking to web pages. This file is identical to the current public release on Sourceforge.net. (ZIP) [file pcbi.1002628.s004.zip › PyDSTool/html/PyDSTool.Redirector-pysrc.html]

xml version="1.0" encoding="ascii"?


PyDSTool.Redirector


| Home | Trees | Indices | Help | PyDSTool |
| --- | --- | --- | --- | --- |

Package PyDSTool ::
Module Redirector

[hide private]

[frames] | no frames]

# Source Code for Module PyDSTool.Redirector

```
 1  """
 
 2      Redirect stdout / stderr to temp file
 
 3  
 
 4   Originally by R. Kern, 2005
 
 5   Adapted by R. Clewley, 2006
 
 6  
 
 7  
 
 8  Copyright (c) 2005 Robert Kern.
 
 9  
 
10  All rights reserved.
 
11  
 
12  Redistribution and use in source and binary forms, with or without
 
13  modification, are permitted provided that the following conditions are met:
 
14  
 
15    a. Redistributions of source code must retain the above copyright notice,
 
16       this list of conditions and the following disclaimer.
 
17    b. Redistributions in binary form must reproduce the above copyright
 
18       notice, this list of conditions and the following disclaimer in the
 
19       documentation and/or other materials provided with the distribution.
 
20    c. Neither the name of the Enthought nor the names of its contributors
 
21       may be used to endorse or promote products derived from this software
 
22       without specific prior written permission.
 
23  
 
24  
 
25  THIS SOFTWARE IS PROVIDED BY THE COPYRIGHT HOLDERS AND CONTRIBUTORS "AS IS"
 
26  AND ANY EXPRESS OR IMPLIED WARRANTIES, INCLUDING, BUT NOT LIMITED TO, THE
 
27  IMPLIED WARRANTIES OF MERCHANTABILITY AND FITNESS FOR A PARTICULAR PURPOSE
 
28  ARE DISCLAIMED. IN NO EVENT SHALL THE REGENTS OR CONTRIBUTORS BE LIABLE FOR
 
29  ANY DIRECT, INDIRECT, INCIDENTAL, SPECIAL, EXEMPLARY, OR CONSEQUENTIAL
 
30  DAMAGES (INCLUDING, BUT NOT LIMITED TO, PROCUREMENT OF SUBSTITUTE GOODS OR
 
31  SERVICES; LOSS OF USE, DATA, OR PROFITS; OR BUSINESS INTERRUPTION) HOWEVER
 
32  CAUSED AND ON ANY THEORY OF LIABILITY, WHETHER IN CONTRACT, STRICT
 
33  LIABILITY, OR TORT (INCLUDING NEGLIGENCE OR OTHERWISE) ARISING IN ANY WAY
 
34  OUT OF THE USE OF THIS SOFTWARE, EVEN IF ADVISED OF THE POSSIBILITY OF SUCH
 
35  DAMAGE.
 
36  
 
37  """ 
38  
 
39  import os 
40  import sys 
41  import tempfile 
42  
 
43  STDOUT = 1 
44  STDERR = 2 
45  
 


46 -class Redirector(object):


47 -    def __init__(self, fd=STDOUT):


48          self.fd = fd 
49          self.started = False

50  
 


51 -    def start(self):


52          if not self.started: 
53              self.tmpfd, self.tmpfn = tempfile.mkstemp(suffix='.pyout') 
54  
 
55              if self.fd == STDOUT: 
56                  self.old = sys.stdout 
57                  sys.stdout = os.fdopen(self.tmpfd, 'w+b') 
58              else: 
59                  self.old = sys.stderr 
60                  sys.stderr = os.fdopen(self.tmpfd, 'w+b') 
61  
 
62              self.started = True

63  
 


64 -    def flush(self):


65          if self.fd == STDOUT: 
66              sys.stdout.flush() 
67          elif self.fd == STDERR: 
68              sys.stderr.flush()

69  
 


70 -    def stop(self):


71          if self.started: 
72              self.flush() 
73              if self.fd == STDOUT: 
74                  sys.stdout.close() 
75                  sys.stdout = self.old 
76              else: 
77                  sys.stderr.close() 
78                  sys.stderr = self.old 
79              tmpr = open(self.tmpfn, 'rb') 
80              output = tmpr.read() 
81              tmpr.close()  # this also closes self.tmpfd 
82              os.remove(self.tmpfn) 
83              self.started = False 
84              return output 
85          else: 
86              return None

87
```

  


| Home | Trees | Indices | Help | PyDSTool |
| --- | --- | --- | --- | --- |

|  |  |
| --- | --- |
| Generated by Epydoc 3.0.1 on Fri May 4 15:24:22 2012 | http://epydoc.sourceforge.net |
